# Exploring transcription modalities from bimodal, single-cell RNA sequencing data

**DOI:** 10.1093/nargab/lqae179

**Published:** 2024-12-18

**Authors:** Enikő Regényi, Mir-Farzin Mashreghi, Christof Schütte, Vikram Sunkara

**Affiliations:** Systems Rheumatology, German Rheumatism Research Centre Berlin, Virchowweg 12, 10117 Berlin, Germany; Visual and Data-Centric Computing, Zuse Institute Berlin, Takustraße 7, 14195 Berlin, Germany; Systems Rheumatology, German Rheumatism Research Centre Berlin, Virchowweg 12, 10117 Berlin, Germany; Modeling and Simulation of Complex Processes, Zuse Institute Berlin, Takustraße 7, 14195 Berlin, Germany; Systems Rheumatology, German Rheumatism Research Centre Berlin, Virchowweg 12, 10117 Berlin, Germany; Visual and Data-Centric Computing, Zuse Institute Berlin, Takustraße 7, 14195 Berlin, Germany

## Abstract

There is a growing interest in generating bimodal, single-cell RNA sequencing (RNA-seq) data for studying biological pathways. These data are predominantly utilized in understanding phenotypic trajectories using RNA velocities; however, the shape information encoded in the two-dimensional resolution of such data is not yet exploited. In this paper, we present an elliptical parametrization of two-dimensional RNA-seq data, from which we derived statistics that reveal four different modalities. These modalities can be interpreted as manifestations of the changes in the rates of splicing, transcription or degradation. We performed our analysis on a cell cycle and a colorectal cancer dataset. In both datasets, we found genes that are not picked up by differential gene expression analysis (DGEA), and are consequently unnoticed, yet visibly delineate phenotypes. This indicates that, in addition to DGEA, searching for genes that exhibit the discovered modalities could aid recovering genes that set phenotypes apart. For communities studying biomarkers and cellular phenotyping, the modalities present in bimodal RNA-seq data broaden the search space of genes, and furthermore, allow for incorporating cellular RNA processing into regulatory analyses.

## Introduction

### Simple mathematical model of transcription

**Figure 1.**
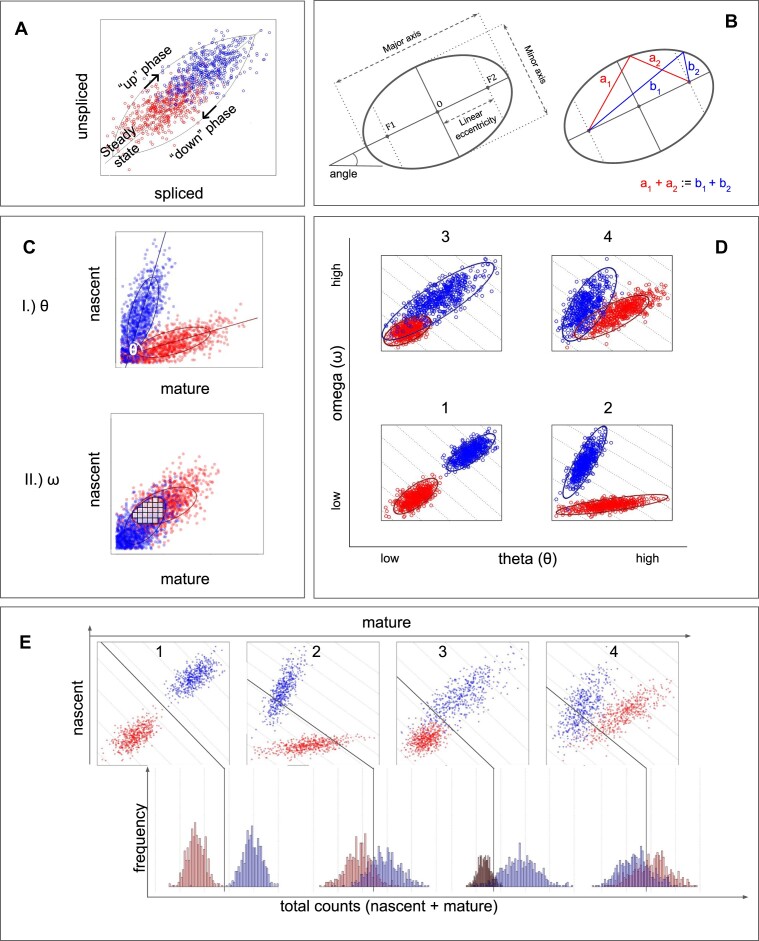
Shapes in splicing-resolved scRNA-seq data. The colours (red and blue) mark different phenotypes under comparison. (**A**) Schematic phase plot demonstrating the basic assumptions of the RNA velocity model, where we highlight the up phase, down phase and steady-state line. (**B**) Anatomy of an ellipse. O denotes the centroid; F1 and F2 are the two focal points. By definition, the sum of distances from the foci to a point on the perimeter is constant. (**C**) Illustration of the two ellipse-derived statistics. I.) Theta (*θ*) is the angle between the major axes of two ellipses. II.) Omega (*ω*) is the area of overlap between two ellipses. (**D**) Demonstration of the ellipse-derived statistics setting apart four different modality types between two phenotypes. (**E**) Schematic phase plots of genes of different modalities and histograms of their count values. The dotted lines marked in the phase plots are lines of nascent = −mature + *c*. The same possible separator lines are marked on the count histograms below. Cells occupying different positions along the same dotted line have an equal count value. The solid lines represent the best separation of the two phenotypes in counts.

It is generally accepted as fact that the transcriptional landscape of a cell is synonymous with its function. Therefore, modelling and analysing transcription is essential to understanding the biology of both health and disease. In 2018, La Manno *et al.* had introduced a system of differential equations to describe the splicing process of messenger RNA and defined RNA velocities in single-cell RNA sequencing (scRNA-seq) (1). This representation had made the picture more dynamic, as it distinguishes between spliced and unspliced transcripts, essentially extracting two consecutive time points instead of just one: spliced transcripts being the presently active ones and unspliced transcripts being the ones that will become active in the near future. We refer to such data as splicing-resolved scRNA-seq data. The mathematical model is formulated by the following ordinary differential equations:


\begin{eqnarray*} \left\lbrace \begin{array}{l}\frac{{\rm d}U}{{\rm d}t} = \alpha - \beta U,\\[4pt] \frac{{\rm d}S}{{\rm d}t} = \beta U - \gamma S, \end{array} \right. \end{eqnarray*}

where *U* denotes the amount of unspliced RNA, *S* denotes the amount of spliced RNA, and *α*, *β* and *γ* are the birth, splicing and degradation rates, respectively. The model is illustrated in Figure 1A.

The model by La Manno *et al.* also accommodates other types of bimodal (or two-dimensional) scRNA-seq data, which are data where two distinct transcript measurements are available for each cell. For instance, splicing-resolved scRNA-seq data provide counts of spliced and unspliced RNA, while time-resolved data capture levels of newly synthesized (new) and pre-existing (old) transcripts. One approach to generate time-resolved data is SH-linked alkylation metabolic sequencing (SLAM-seq), where the incorporation of metabolic labels over time enables the quantification of old and new transcripts (2). By substituting these measurements for spliced and unspliced counts in the above equations, the resulting velocity will remain meaningful and reflect RNA turnover rather than splicing dynamics.

Currently, RNA-seq research is focused on finding out whether the total expression changes between two or more conditions, evidenced by the number of tools and methods available for this purpose (3). Induction or repression of a gene can be interpreted as a switching on or off of transcription, meaning a change in *α*. This approach, however, overlooks changes in the splicing and degradation rates, *β* and *γ*, that also do occur and are of biological importance. One example of this is RNA interference (RNAi). RNAi is a naturally occurring cellular machinery, universally found in both mammalian and non-mammalian systems, and can alter splicing or silence messenger RNA post-transcription (4,5). As the development and application of RNA-based therapeutic approaches is on the rise, RNAi-based methods are receiving particular attention (6). This is in part due to their unique ability of acting at a transcript level. This is desired, as transcriptional regulation plays an important role in many—if not all—malignancies, including tumorigenesis and neurodegenerative, cardiovascular and infectious diseases (7). Screening for and finding changes in endogenous RNA processing is therefore crucial and can be achieved by defining and applying useful mathematical models of transcription.

### Dynamic versus static approach

In the RNA velocity model (see the ‘Simple mathematical model of transcription’ section), it is assumed that each cell is a snapshot in time as it traverses along a fixed trajectory. When fitted, the interpretation of the model parameters is naturally biased to the transcriptional complexity that the model assumes (8). Furthermore, in the RNA velocity model, the focus is strictly on capturing the transitions between phenotypes with no replicates accounting for noise. In this work, we move away from the notion of velocities and dynamics. Our goal is to parametrize scRNA-seq data such that it becomes possible to contrast phenotypes in the two-dimensional space, rather than exploring the transitions between them. We propose a static approach that, similarly to differential gene expression analysis (DGEA), treats cells within a phenotype as biological replicates. DGEA is analysing the total expression, missing the transcriptional modalities apparent in the two-dimensional counts. To expend this classical notion, we propose characterizing the data as ellipses, the simplest of parametric models, and studying differences between phenotypes in a two-dimensional space.

### Ellipse parametrization of transcription

The usefulness of a model is always limited by the availability of data sufficient in both quality and amount. Today, scRNA-seq is routinely generated in many research projects, providing a resolution and abundance of data that was unheard of in the era of bulk sequencing. Furthermore, information on whether the captured transcript is nascent or mature is also available, providing two dimensions to expression, instead of just one (total expression). This increased dimensionality of the data—compared to regular RNA-seq—is not yet exploited to the fullest. When determining the significance of a gene, only one dimension is considered, the total expression, via DGEA. Higher order structures that may indicate subtle changes in regulation are not yet widely investigated.

Defining useful mathematical models of transcription is an ongoing effort in the field of computational biology (1,9–14). New models may be more efficient or more accurate than their predecessors, or may explore previously unexplored aspects of the data. Contributing to that effort, and aiming for the latter, we propose using ellipses as a parametrization of two-dimensional scRNA-seq data. Gene expression in RNA-seq data most frequently follows a normal distribution (15). When working with both nascent and mature counts, this translates to a multivariate normal distribution, which is inherently elliptical. Therefore, ellipses are not just higher order, distribution-free parametrizations of scRNA-seq data, but also inherently present in the structure.

An ellipse is a curve around two focal points, such that given any point on that curve, the sum of its distances to the focal points is constant (see Figure 1B). Mathematically, it is described by a specific section of a cone, and with the equation


\begin{eqnarray*} ax^2 + bxy + cy^2 + dx + ey + f &= 0, \end{eqnarray*}

with the coefficients satisfying the following constraint:


\begin{eqnarray*} 4ac - b^2 < 0 . \end{eqnarray*}

### Ellipse-derived statistics

The original model of RNA velocity includes the definition of a steady-state line. This is a linear fit to the data points, going through the origin, such that the angle of this line is an approximation of *β*/*γ*. Analogous to the steady-state line, we study the major axis of the ellipse fit. More importantly, we define the angle difference between the major axes of ellipses fit on two phenotypes (looking at the same gene) and denote this by the Greek letter *θ* (see Figure 1C-I). We interpret a change in the angle *θ* as an indicator of altered physiological RNA processing, such as a shift in splicing or degradation rates. Therefore, we suspect that the occurrence of a large *θ* is indicative of important biological and regulatory processes, similarly to a change in total expression. Thus, we use *θ* as a statistic for detecting such changes.

Another statistic that we derive from the ellipse representation is the overlap, which we denote by the Greek letter *ω* and define as the Sørensen–Dice coefficient between the area of two ellipses (16,17). In Figure 1C-II, we illustrate how *ω* is estimated. The biological interpretation of this statistic is the following: If the ellipses fit on two conditions are overlapping to a high degree, expression and transcription dynamics are expected to be similar. If the overlap is low, the gene may be differentially expressed between the two conditions or may display differential transcription dynamics.

The two statistics together, the angle *θ* and the overlap *ω*, define four possible modalities: small overlap and small angle difference (scenario 1), small overlap and large angle difference (scenario 2), large overlap and small angle difference (scenario 3), and lastly, large overlap and large angle difference (scenario 4) (see Figure 1D). As DGEA operates with total counts, it only detects scenarios 1 and 3, as demonstrated by Figure 1E.

### Transcriptional regulation

Literature suggests that the splicing rate plays an important role in cell biology (1,18,19). The duration between the beginning of transcription and the completion of a mature transcript can range from a few minutes to hours. Thus, the proliferation rate of cells appears to shape the transcriptional landscape, which may be only one of the ways splicing is exploited for regulation (20). As another example, co-transcriptional splicing is linked to 3′ end cleavage. The experiments of Reimer *et al.* (21) indicate that co-transcriptional splicing efficiency determines 3′ end processing efficiency. It was also found that histone modifications play various roles in splicing (22,23). In addition to splicing kinetics, RNA degradation plays a critical role in regulating RNA stability and overall transcript levels (24).

The above studies suggest that RNA processing plays a critical role in transcriptional regulation, yet it remains unexplored. As argued for by Alpert *et al.* (18), more research is needed to determine the exact mechanisms of splicing in the context of regulation. Hence, we propose detecting the phenomena as captured by two-dimensional scRNA-seq data.

### Summary of the present work

In the present work, we use ellipse representations of gene expression in bimodal scRNA-seq data. We derive two statistics from the ellipse fits, the angle *θ* and the overlap *ω*. We use these statistics to describe four different transcriptional modalities present in the two-dimensional space. We further investigate gene modalities of a high *θ* and term them differentially angled genes (DAGs). DAGs do not always show up in DGEA; however, they do discriminate between two phenotypes. These differences suggest potential biological significance; however, further investigation is needed to confirm and determine their exact role in biological processes and conditions.

## Materials and methods

### Software

For analysis, R v4.1.1 (25) and R studio v2022.07.1+554 (26) were used. For code availability, please refer to the ‘Code availability’ section.

### Data preprocessing

For data availability, please refer to the ‘Data availability’ section.

We used DropletQC (27) with its default settings to remove doublets and burst cells, as well as manual thresholds for minimum and maximum library size. We determined the manual thresholds after inspecting the distribution and elbow plots of the total counts per cell. For the cell cycle (CC) dataset, the minimal and maximal total count values were set to 40 000 and 150 000, and for the colorectal cancer (CRC) dataset, 2000 and 50 000. An additional threshold was used for the CRC dataset: a minimal variance in counts of 3, to narrow the search space. No smoothing was applied. Normalization to library size was done by dividing each count value by the total count of its corresponding cell and multiplying by mean library size. The mean library size was calculated using only viable cells, meaning after application of the thresholds described earlier.

Prior to ellipse fitting, we applied additional criteria, to avoid singularity when solving for the least-squares ellipse fit. First, we selected genes where both phenotypes were expressed with a mean count of >5. As the CC dataset had only around 300 cells in each batch, we further checked whether there were 20 or more non-zero cells in both phenotypes for each gene in each batch. Then, in a gene-wise manner, cells whose nascent, mature or total counts were 0 were removed. Following that, nascent and mature reads were independently min–max normalized. Outliers were removed in each phenotype by excluding cells above the 99 percentile of both nascent and mature counts. Finally, the data were slightly jittered using the R base jitter() function with default parameters. The visualizations in the paper are not displaying jittered data.

### Cell cycle scoring

To identify the cell cycle phase in the CC dataset, we used Seurat v4.1.1 (12) to score the cells. The algorithm assigns probabilities to a cell being in either G2M or S phase, based on the expression of the marker genes prescribed in the package. We then took cells that were in the top 25% of G2M scores/probabilities and labelled them as G2M. We did the same for the phase S scores. The rest of the cells were labelled as ambiguous and were not used for subsequent differential analyses performed on the CC dataset.

### Differential gene expression analysis

For DGEA, we used Seurat v4.1.1 (12) for both the analysis and the preprocessing. Seurat’s built-in functions were used to normalize and scale the data, as advised by its user manual. DGEA scores were computed using FindAllMarkers() with setting test.use = ‘DESeq2’. Genes were considered significant if they had an absolute log_2_ fold change (LFC) of ≥1, an adjusted *P*-value [or false discovery rate (FDR)] of ≤0.01 and a mean expression of >10. We drew conservative thresholds to ensure that the differentially expressed genes (DEGs) we consider are very distinct between the two phenotypes. Finally, we defined recurrent DEGs as ones shared by all four runs (batches 1–3 and the pooled data) in the CC dataset and by both patients in the cancer dataset.

### Ellipse fitting

For ellipse fitting, we used the numerically stable Fitzgibbon approach, outlined below.

An ellipse can be defined by the equation


\begin{eqnarray*} ax^2 + bxy + cy^2 + dx + ey + f &= 0, \end{eqnarray*}

with coefficients *a*, *b* and *c* satisfying the following constraint:


\begin{eqnarray*} 4ac - b^2 < 0 . \end{eqnarray*}

A convenient way of fitting an ellipse is using a numerically stable extension of the Fitzgibbon approach, presented by Halır and Flusser (28,29). This approach is robust, easy to implement and efficient. Briefly, the goal is to find the coefficient vector **a**, for which the sum of distances of the data points to the conic is minimal. We can define the vector of coefficients, **a**, and the vector of variables, **x**, as follows:


\begin{eqnarray*} {\bf a} &:= [ a, b, c, d, e, f ]^{\rm T} \end{eqnarray*}

and


\begin{eqnarray*} {\bf x} &:= [ x^2, xy, y^2, x, y, 1]. \end{eqnarray*}

Then, the generic conic equation can be written as


\begin{eqnarray*} F_{{\bf a}}(x) := {\bf x}\cdot {\bf a} = 0, \end{eqnarray*}

and then the minimization problem of fitting an ellipse to *N* data points can be formulated as


\begin{eqnarray*} \underset{{\bf a}}{\text{min}} \sum _{i=1}^{N}(F_{{\bf a}}(x_{i}))^2. \end{eqnarray*}

With the ellipse-specific constraint, and considering that an arbitrarily scaled parameter vector represents the same ellipse, the above minimization problem reformulates to


\begin{eqnarray*} \underset{{\bf a}}{\text{min}} \Vert D{\bf a}\Vert ^{2}, \end{eqnarray*}

subject to


\begin{eqnarray*} {\bf a}^{{\rm T}}C{\bf a} = 1, \end{eqnarray*}

where *D* is the design matrix, filled with all linear combinations of the independent variables *x* and *y* according to the generic conic equation, and *C* is the constraint matrix:


\begin{eqnarray*} D &:= {\begin{pmatrix}x^2_{1} & x_{1}y_{1} & y^2_{1} & x_{1} & y_{1} & 1\\ \vdots & \vdots & \vdots & \vdots & \vdots & \vdots \\ x^2_{i} & x_{i}y_{i} & y^2_{i} & x_{i} & y_{i} & 1\\ \vdots & \vdots & \vdots & \vdots & \vdots & \vdots \\ x^2_{N} & x_{N}y_{N} & y^2_{N} & x_{N} & y_{N} & 1 \end{pmatrix}} \end{eqnarray*}

and


\begin{eqnarray*} C &:= {\begin{pmatrix}0 & 0 & 2 & 0 & 0 & 0 \\ 0 & -1 & 0 & 0 & 0 & 0 \\ 2 & 0 & 0 & 0 & 0 & 0 \\ 0 & 0 & 0 & 0 & 0 & 0 \\ 0 & 0 & 0 & 0 & 0 & 0 \\ 0 & 0 & 0 & 0 & 0 & 0 \end{pmatrix}}. \end{eqnarray*}

We can then introduce the scatter matrix, or matrix of moments, *S*, with


\begin{eqnarray*} S := D^{{\rm T}}D = {\begin{pmatrix} S_{x}^{\,\,4} & S_{x}^{\,\,3}{}_y & S_{x}^{\,\,2}{{}_y}^{\,\,2} & S_{x}^{\,\,3} & S_{x}^{\,\,2}{{}_y} & S_{x}^{\,\,2} \\[4pt] S _{x}^{\,\,3}{}_{y} & S _{x}^{\,\,2}{}_{y}^{\,\,2} & S _{x}{}_{y}^{\,\,\,3} & S _{x}^{\,\,2}{}_y & S _{x}{}_{y}^{\,\,\,2} & S _{x}{_y}\\[4pt] S _{x}^{\,\,2}{}_{y}^{\,\,2} & S _{x}{}_{y}^{\,\,\,3} & S _{y}^{\,\,4} & S _{x}{}_{y}^{\,\,2} & S _{y}^{\,\,3} & S _{y}^{\,\,2} \\[4pt] S _{x}^{\,\,3} & S _{x}^{\,\,2}{}_{y} & S _{x}{}{_y}^{\,2} & S _{x}^{\,\,2} & S _{x}{}_{y} & S _{x} \\[4pt] S _{x}^{\,\,2}{}_{y} & S _{x}{}_{y}^{\,\,2} & S _{y}^{\,\,3} & S _{x}{}_{y} & S _{y}^{\,\,2} & S _{y} \\[4pt] S _{x}^{\,\,2} & S _{x}{}_{y} & S _{y}^{\,\,2} & S _{x} & S _{y} & S _{1} \end{pmatrix}} \end{eqnarray*}

with


\begin{eqnarray*} S_{x}^{\,\,a}{}_{y}^{\,\,\,b} &= \sum \limits _{i=1}^{N} x _{i}^{\,\,a}y _{i}^{\,\,b}. \end{eqnarray*}

Utilizing Lagrange optimization, we can formulate the constraints as


\begin{eqnarray*} S{\bf a} = \lambda C{\bf a}\quad \text{ and } \quad{\bf a}^{{\rm T}}C{\bf a} = 1. \end{eqnarray*}

Then, the optimal solution is the eigenvector **a**_*k*_, corresponding to the minimal positive eigenvalue, *λ*_*k*_, of the system


\begin{eqnarray*} \Vert DA\Vert ^{2} = {\bf a}^{{\rm T}}D^{{\rm T}}D{\bf a} = {\bf a}^{\rm T}{\bf a} = \lambda {\bf a}^{{\rm T}}C{\bf a} = \lambda . \end{eqnarray*}

### Implemented ellipse fitting strategies

We implemented two approaches for fitting ellipses in the accompanying code: (i) freely fitting ellipses to minimize root mean square deviation (RMSD) and (ii) fitting ellipses with their major axis forced through the origin. Approach (i) provides a fit that aligns more closely with the data, while approach (ii) enforces alignment with the assumptions of the RNA velocity model. The default setting minimizes RMSD by fitting ellipses freely. Supplementary Figure S7 demonstrates the two approaches, including RMSD values for each fit.

### Bootstrapping ellipse fits

The ellipse fit of each gene and each phenotype was bootstrapped 1000 times, and to avoid the singularity of the matrix of moments, the data were jittered with *ε* ≤ 0.5.

### Gene filtering with respect to ellipse fits

To see how well the ellipses fit to the data, we have calculated the RMSD of the fits (see Supplementary Figure S6). Above a threshold, the error stabilizes. We found that very low expression genes are typically not good candidates for this analysis.

The RMSD was computed by taking the average of the absolute distance of each point after min–max normalization (but no jittering) to the perimeter of the ellipse fit.

### Overlap (*ω*) and angle (*θ*) calculation

The overlap *ω* was defined as the Sørensen–Dice coefficient between ellipses fit onto two phenotypes (16,17). The Dice coefficient in simple terms is two times the area of intersection divided by the total area or union of the two ellipses.

While there is a straightforward formula for calculation of the area of an ellipse, calculating the area of intersection between two ellipses is numerically challenging. We approximate the area of intersection using the box counting method. To keep area calculations consistent, we use the same method for computing the area of the ellipses as well.


\begin{eqnarray*} \phi = 12\cdot {\rm atan} \left( \frac{2b}{a-c} \right). \end{eqnarray*}

*θ* was calculated as the difference between the major axis angles of two phenotypes:


\begin{eqnarray*} \theta := \left| \phi _{1} - \phi _{2} \right|. \end{eqnarray*}

For differential angle analysis, relevant genes were defined as ones having a major axis length of >0.1 for the CC dataset and 0.01 for the CRC dataset, a *θ* value of >10° and <160°, a mean expression of >10 and an absolute LFC of <1. These thresholds were chosen considering that (i) we were mostly looking for genes where two phenotypes expressed at a comparable scale, but (ii) had a difference in angle, and (iii) fits with a very small major axis or low expression may not be as reliable. As a final measure of relevancy, we defined recurrent DAGs as ones shared by all four runs (batches 1–3 and the pooled data) in the CC dataset and by both patients in the cancer dataset.

The resulting genes were each assigned a rank and sorted in a descending order. The rank was based on the ratio of the major and the minor semi-axis; more precisely, for each gene, the rank was calculated by taking all fits over all batches or patients and finding the minimal semi-axis ratio. This rank served as a proxy for our confidence in the fit. A large ratio means high ellipticity, in which case the angle is more likely to be robust. A low ratio is indicative of a fit close to a circle, which means that small differences in the data can change the direction of the major axis.

### Description of data

#### CC dataset

There were three batches in this dataset. We ran our pipeline on each batch separately, as well as on all cells from all batches, pooling the data. Batches 1–3 included 645, 400 and 323 cells, respectively, 1368 in total, and 10 050 non-zero genes.

#### Cancer dataset

The raw dataset included 21 759 cells assigned to organoids healthy, patient 009 and patient 013, of which 6974 cells passed the quality control (QC) measures and were marked viable. Of those 6974 cells, 1865 were healthy, 2393 belonged to patient 009 and 2716 belonged to patient 013. In total, 25 238 genes met the variance threshold.

### Gene Ontology enrichment analysis

Gene Ontology (GO) enrichment was performed using geneontology.org (30,31) for human biological processes on the lists of DEGs and DAGs.

### Gene set enrichment analysis

Gene set enrichment analysis (GSEA) was performed using the GSEA software (32,33) against the C6 collection (oncogenic signature gene sets) from the Molecular Signatures Database (34,35). GSEA was performed twice on all expressed genes, once ordered by −log_10_(FDR + 0.001) × LFC, as obtained from DGEA, and once by *θ* × rank, as obtained from the ellipse fits, where *θ* is in pi radians. After ordering, the gene sets were analysed using the ‘Preranked’ GSEA tool.

### Code availability

R code for implementing the ellipse representation and deriving the statistics *θ* and *ω* is provided as supplementary information. The code is available at our GitHub repository (https://git.zib.de/sunkara/ellipses-for-transcription-modality-discovery).

## Results and discussion

### Highly expressing genes exhibit ellipticity

Our goal was to model two-dimensional scRNA-seq data to enable the extraction of modalities. We chose ellipses, because they fit the shape of the data and are well-understood geometrical objects.

To assess whether an ellipse is useful for modelling bimodal expression data, we used multiple datasets to fit our model onto different phenotypes in a gene-wise manner. We then determined the modalities of the genes using ellipse-derived statistics, the overlap (*ω*) and the angle (*θ*). We checked whether these genes also appeared in DGEA based on total counts. We did this to investigate the added benefits of using our method in conjunction with this well-established, widely used approach. Our findings indicate that using ellipses identifies genes that show distinct modalities between phenotypes. Thus, we define the notion of DAGs. While these differences could be due to changes in transcription dynamics, further validation is required to confirm their biological impact. In the following subsections, we go through our results, dataset by dataset, and discuss our findings.

#### CC dataset

**Figure 2.**
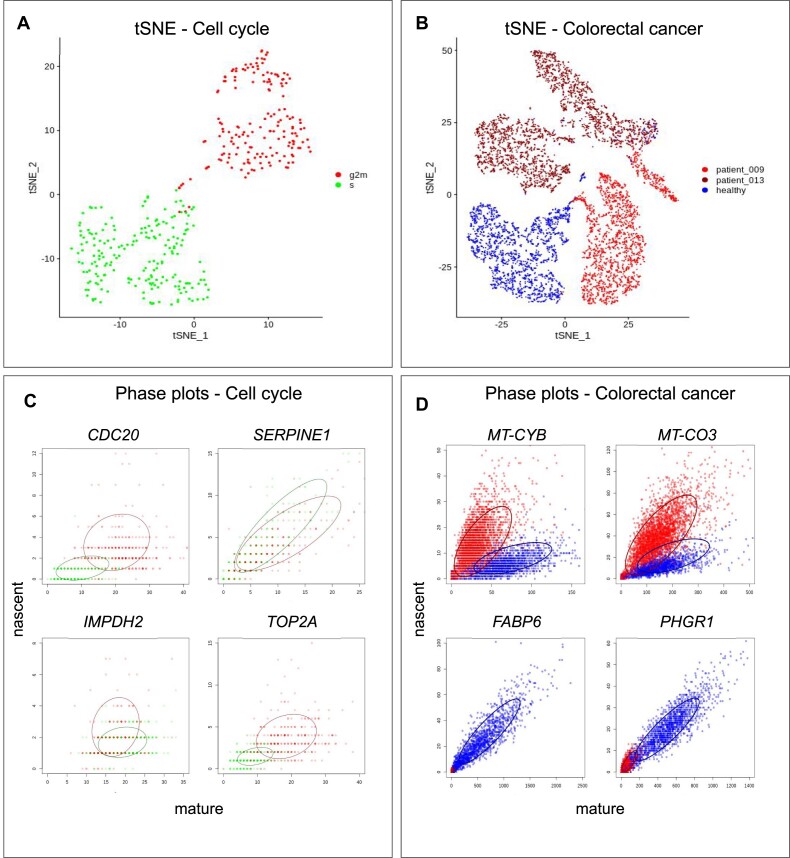
DEGs and DAGs are present in the CC and CRC datasets. (**A**) t-distributed stochastic neighbor embedding (t-SNE) plot of the CC dataset. Cells in phase S are marked in green, and cells in phase G2M are marked in red. (**B**) t-SNE plot of the CRC dataset. Healthy cells are marked in blue, and cancer cells are marked in red (patient 009) and dark red (patient 013). (C, D) The *x* and *y* axes in the scatter plot are counts of mature and nascent RNA, respectively. (**C**) Phase plots of *CDC20*, *SERPINE1*, *IMPDH2* and *TOP2A* genes in the CC dataset, visualized using all batches (pooled), and the corresponding ellipse fits in solid lines. (**D**) Phase plots of *MT-CYB*, *MT-CO3*, *FABP6* and *PHGR1* genes in the CRC dataset, in patient 013, and the corresponding ellipse fits.

The first dataset used was of U2-OS cells, a CC dataset (see Figure 2A). The dataset contains three batches. No batch effect was observed (see Supplementary Figure S1). We labelled the cells to be in either G2M or S cycle phase, and fit ellipses to these phenotypes separately, on a per gene basis. We repeated our analysis on all batches separately, as well as on data pooling all batches. We compared cells in cell cycle phases G2M and S, and found 41 recurrent DEGs and 119 recurrent DAGs, where recurrent genes are defined as ones that were either significant (absolute LFC > 1 and FDR < 0.01) or relevant (a major axis length of >0.1, a *θ* value of >10° and <160°, a mean expression of >10 and an absolute LFC of <1) in all four experiments. Given that the definition of DAGs requires an |LFC| < 1, there are no overlapping genes between DAGs and DEGs. We then ranked the expressed genes according to their ellipticity and major axis angle between phenotypes and investigated the ranking. Based on the elbow of the gene ranks, ultimately, the top 20 genes were selected. We found that the modalities described earlier (Figure 1D and E) are present and detectable in the data (Figure 2C).

We performed GO enrichment on both the DEGs and the DAGs. The top 30 of all significant GO terms are presented in Supplementary Figure S2. The DEG set’s most significant terms were all cell cycle related, with the term ‘cell cycle’ having an FDR of 3.79 × 10^−18^. However, there was no significant enrichment when analysing DAGs. This outcome was to be somewhat expected, as biological processes had always been studied in terms of differential expression; following from that, databases may simply not have annotation related to the type of change our method is detecting.

**Figure 3.**
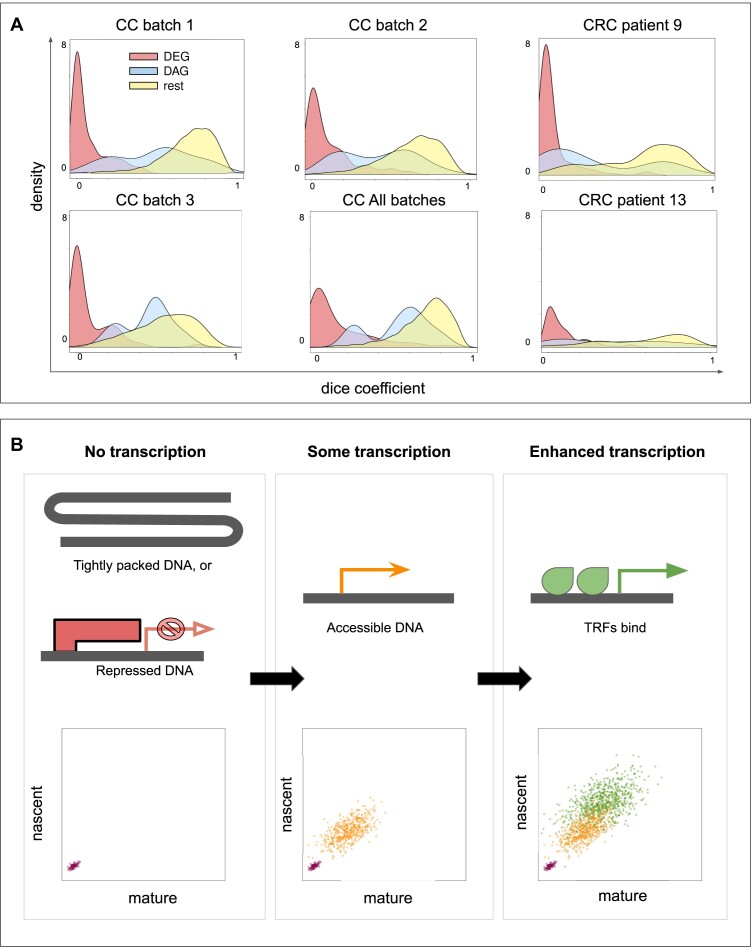
Differences in transcription modalities are indicated by the overlap statistic *ω*. (**A**) Frequency distribution of *ω* in DEGs (red), DAGs (blue) and non-relevant genes (rest, yellow). All three batches (separately and pooled) of the CC dataset and both patients of the CRC dataset are shown. (**B**) (Top) Cartoon illustrating three biological states of a gene: inaccessible, accessible and induced by transcription factor binding. (Bottom) The corresponding expected expression profiles in the phase plot: no transcription, some transcription and enhanced transcription, respectively.

The second statistic we defined is the overlap *ω*. DGEA by definition picks up genes whose count distribution is distinct between two phenotypes, which in the context of ellipses can only occur when *ω* is also low, as illustrated by Figure 1E. In DAGs, we expect that *ω* is larger than that in DEGs. In practice, we expect that the larger *θ* is, the smaller *ω* is. Non-relevant genes are defined by the two phenotypes having an identical distribution; therefore, a high overlap is to be expected. *ω* had shown a pattern that was consistent in all four experiments. The statistic’s distribution when considering DEGs, DAGs or the rest of the genes (non-significant population) has a clear trend to lower, mid-range and high overlap, respectively, as demonstrated by Figure 3A.

#### Cancer dataset

The second dataset comprised time-resolved scSLAM-seq data from organoids derived from healthy colon tissue and CRC samples from two patients (see Figure 2B). We performed two experiments on the cancer dataset, following the same protocol as before in identifying the DEGs and DAGs. The raw dataset included 21 759 cells assigned to organoids (healthy, patient 009 or patient 013) and 25 259 genes in total. A total of 6974 cells passed the QC measures. A total of 1865 cells belonged to the healthy organoid, 2393 belonged to the organoid derived from patient 009 and 2393 belonged to the organoid derived from patient 013. After performing our analysis, 96 recurrent DEGs and 17 recurrent DAGs were found. The DAGs were assigned a rank based on the ellipticity of the fit and they were ordered. These 17 DAGs were considered relevant.

Some examples of such genes that display different modalities are shown in Figure 2D. After ranking and ordering all expressed genes, as explained in our methods, we ran GSEA on both ranked lists using a collection of oncogenic gene sets.

In both patients, when looking at the GSEA of ranked DAGs, the FDR values were non-significant (${\ge} 25\%$). However, when running the analysis on the list of genes ranked by the DGEA metrics, there are numerous oncogenic gene sets significantly enriched (see Supplementary Figures S3 and S4). As observed with the CC dataset, the database annotation of DAGs alone is not associated with the phenotype we are detecting them from.

To see whether there is documentation of the DAGs in the context of cancer, we investigated some of them manually. Looking at the recurring DAGs, several mitochondrial genes are highlighted. This is reassuring, considering the fact that the mitochondria had been studied for years in the context of tumorigenesis and metastasis, and some mitochondrial genes had even been identified as important biomarkers (36–38). One of the recurrent DAGs found with our methods, *MT-CYB* (Figure 2D), is notably implicated in multiple cancer types, including CRC (39). *MT-ND1* (Supplementary Figure S5B), as another example, had been found to have multiple mutations in CRC (40). Other mitochondrial DAGs are not confirmed as oncogenes to our knowledge. An example of that is *MT-CO3* (see Figure 2D).

In all of the above genes, overall, the angle of new and old transcripts decreases in cancer. In other words, the relative amount of new transcripts is increased. Given that the change in the total amount of RNA is not striking, these observations imply that transcripts of such genes may go through their life cycle faster.

Using our shape-based method, more genes, in addition to DEGs, could be discovered that set apart healthy and cancerous phenotypes. Some, as mentioned earlier, are already linked to CRC, but some remain to be confirmed as potential biomarkers and/or participants in cancer pathology.

#### Summary of analyses

Ellipses were possible to fit over multiple datasets and multiple batches. Ellipse-derived statistics (the angle *θ* and the overlap *ω*) enable highlighting genes of novel modalities in multiple experiments. The distribution of said statistics follows our theoretical expectations. The fitting error stabilizes above a certain expression level (see Supplementary Figure S6). We were able to identify several DEGs and DAGs in both the CC and the CRC dataset. The GO enrichment and the GSEA have revealed that while databases are immensely useful for DGEA studies, they currently do not necessarily include annotation on genes of the novel modalities we are detecting. We took a closer look at DAGs and tried to relate them to the phenotypes. We found that there is an overlap with our current knowledgebase.

It is also to be noted that different biological processes might show different signals. A cell cycle process is a closed, smooth loop with minor changes in expression along the trajectory, while cancerous growth results in a differentiation-like process, where a cell loses and/or gains characteristics. In alignment with this, we saw more pronounced differences in the cancer dataset than in the CC dataset.

To prove the biological relevance of the DAGs beyond dispute, wet lab experiments would have to be carried out, which are beyond the scope of this bioinformatics research paper. However, inspection of the two-dimensional counts of the genes in question reveals visible distinctions between two phenotypes (e.g. *MT-CYB* and *MT-CO3* in Figure 2D). The change in angle of DAGs is as distinct as the change in expression of DEGs. Furthermore, deviation in the complex biology of cells is rarely without effect. Based on our results, the shape information reveals a type of difference between phenotypes that is yet to be studied in more detail.

### DEGs are following two modality types

We found that DEGs mainly follow two modalities out of the four we expected that genes would. The first of those modalities (scenario 1 in Figure 1D) is a striking on/off switch, where the gene is expressed on a large scale in one phenotype, and barely or not at all in the other. In terms of the ellipse statistics, there is no angle change and no overlap (e.g. see *FABP6* in Figure 2D). The second modality a differentially expressed gene follows (scenario 3 in Figure 1D and E) also involves an increase of expression, but it is a switch from a lower, non-zero expression profile to a higher expression profile. The difference in angles is again negligible, but there is an overlap of varying degrees between the two phenotypes (e.g. see the gene *CDC20* or *PHGR1* in Figure 2C and D).

Furthermore, the angle differences were low and similar to the angle differences measured in non-relevant genes. In non-relevant genes, we expect the cells of the two compared phenotypes to be distributed similarly, have the same angle and a high overlap. There was no decisive trend indicating a difference between the *θ* values of DEGs and non-relevant genes. To test this, we used a two-sided Wilcoxon rank-sum test, the results of which are given in Table 1.

**Table 1.** Two-sided Wilcoxon test results comparing the *θ* values of DEGs and non-relevant genes

	# DEGs	# Non-DEGs or non-DAGs	*U* statistic	*P*-value	95% CI
CC, batch 1	84	5231	205 364	0.3041	[−8.702567, 2.263026]
CC, batch 2	106	4893	210 106	0.000813*	[−16.21031, −3.54580]
CC, batch 3	116	3271	164 268	0.01394*	[−16.210122, −1.448104]
CC, all batches	102	5654	241 416	0.004775*	[−10.080555, −1.260748]
CRC, patient 009	200	251	24 320	0.5708	[−6.987461, 2.188695]
CRC, patient 013	196	282	20 938	0.00006512*	[−21.758386, −5.130871]

Columns state the number of DEGs, the number of non-DEGs with DAGs removed, the *U* statistic of the *θ* values of these genes, the significance of the results (*P*-value), with * indicating a *P*-value ≤0.05 satisfied, and the 95% confidence interval (CI).

There are many possible biological interpretations of DEGs following the two modalities discussed earlier. One might be that if a repressed or inaccessible gene becomes accessible and is transcribed, then we see a sudden appearance of expression that could be viewed as a switching on of that gene. However, when a gene is already accessible to begin with and is transcribed to some degree, the increase in expression could be due to another process, e.g. binding of additional transcription factors. Hence, we see now an increase in total expression, but we expect no change in splicing or turnover (see Figure 3B).

### Ellipse-derived statistics detect previously overlooked modalities

After DGEA, we fit ellipses and derive the angle *θ* and the overlap *ω* between phenotypes. Using the angles, it is possible to detect genes where the phenotypes demonstrate a clear separation in two dimensions, which is not visible in the one dimension of total counts. The first new modality we detect this way can be described by a high angle difference and low overlap (see scenario 2 in Figure 1D or gene *MT-CYB* in Figure 2D). The second, with an angle difference, and some more overlap between phenotypes (see scenario 4 in Figure 1D or gene *SERPINE1* in Figure 2C and gene *MT-CO3* in Figure 2D). The distribution of the overlap statistic of DAGs is wide (see Figure 3A).

We were able to extract genes that visibly differentiate two phenotypes, possibly due to a difference in transcription dynamics. DAGs appear to differentiate cancerous and healthy conditions more prominently, which could be due to the fact that cells in different cell cycle phases are more similar to each other than healthy and cancer cells. Notably, the changes in DAGs that we detect do not result in significant changes to the total expression. We are reporting on and exploring this new type of phenotypic difference that is visible in two-dimensional scRNA-seq data. Biology rarely produces patterns without function or purpose; therefore, we believe that investigation of DAGs could uncover new insights, for example possible regulatory shifts, as changes in the endogenous processing of RNA leave opportunity for regulation. Future validation and exploration of these novel modalities and their biological impact should include wet lab experiments. For example, as some DAGs in the cancer dataset were mitochondrial genes, it could result in a measurable difference in oxygen consumption rate between healthy and cancerous cells. Along the same lines, mitochondrial stress assays could also be employed to measure how cells respond to mitochondrial stress in terms of their ability to recover or survive. More gene-specific techniques include CRISPR assays and hybridization-based knock-down methods. It could be desirable to only partially enhance or silence the target gene, to mimic the effect visible in the data better.

### Alignment with current literature

Two recent studies had also explored the two-dimensional transcription space of single cells. Carilli *et al.* had investigated transcriptional and splicing dynamics, and had found distributional differences between certain cell subclasses that did not result in mean expression changes, and stipulated that this was due to differential regulation (41). This finding is in alignment with the results of our model; for example, modalities 2 and 4 in Figure 1E may not show a mean expression change between the two phenotypes, yet in two dimensions, there is a difference in the distribution of cells. Another study by Volteras *et al.* had identified genes whose kinetic rates were cell cycle phase dependent (42). These findings align with our results, as phenotype-dependent kinetic parameters would result in differences between groups of cells in the two-dimensional transcription space, while not necessarily cause mean expression changes.

### Currently standard preprocessing distorts shape information

Lastly, we would like to draw attention to an important observation we made when trying different preprocessing and filtering methods. Since scRNA-seq data tend to be noisy, most standard pipelines apply some smoothing method, *K* nearest neighbour (KNN) smoothing being the most popular (1,12,43,44).

**Figure 4.**
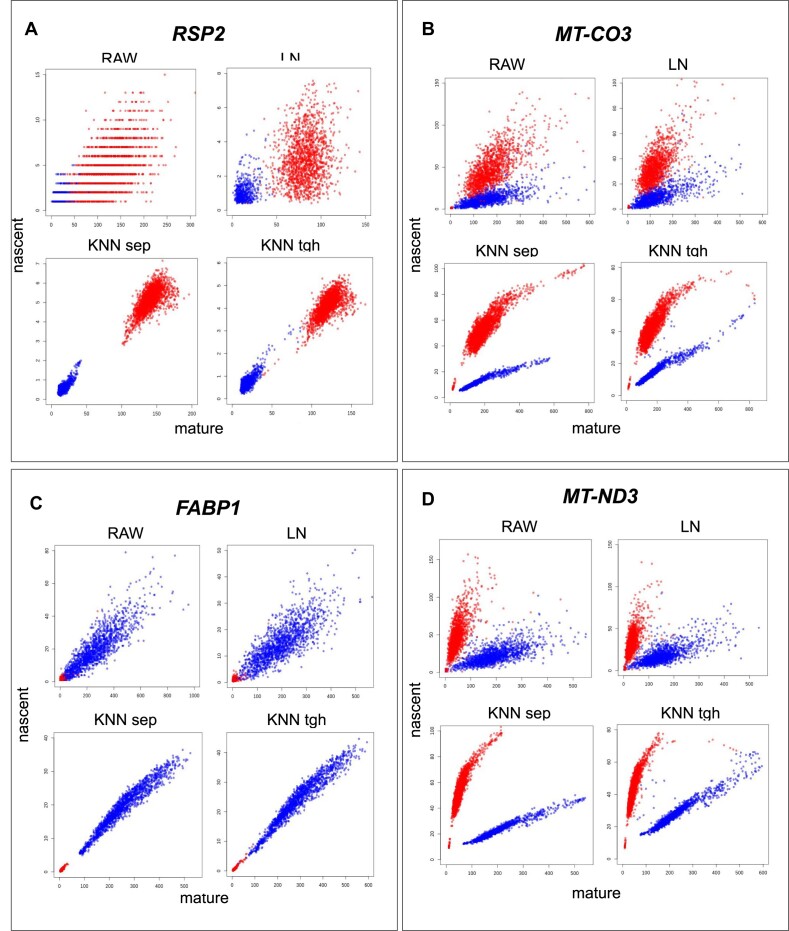
(**A**–**D**) Effect of normalization and smoothing demonstrated on genes *RSP2*, *MT-CO3*, *FABP1* and *MT-ND3* from the CRC dataset, respectively. Within each panel, the effect of the following preprocessing methods is shown: (RAW) phase plot of raw counts, (LN) counts normalized to library size (library normalized), (KNN sep) KNN smoothing separately, per phenotype, and (KNN tgh) KNN smoothing on all cells. Blue: healthy tissue; red: cancerous tissue.

Figure 4A–D displays the effect of smoothing on genes *RSP2*, *MT-CO3*, *FABP1* and *MT-ND3* in the CRC dataset. Panels RAW, LN, KNN sep and KNN tgh correspond to phase plots of the raw data, the data normalized to library size, the data after per-phenotype (separate) KNN smoothing and KNN smoothing all cells together. The modalities of *FABP1*, *MT-ND3*, *RSP2* and *MT-CO3* correspond to scenarios 1–4, respectively, in Figure 1D and E. Normalization to library size is a logical, even necessary step, as differing sequencing depths need to be accounted for when comparing phenotypes. Smoothing, however, is a denoising step.

We observe that scaling does not fundamentally change the shape of the data and the relation of the phenotypes to each other. KNN smoothing makes the data more compact in all cases. KNN smoothing either per phenotype or on all cells together increases the separation of the two phenotypes in the counts of *FABP1* and *RSP2*. We also observe that smoothing ‘thins’ the data cloud, pulling the cells towards the midline. This destroys the overlap *ω* between the two phenotypes. In case of *MT-ND3* and *MT-CO3*, KNN smoothing, besides thinning the data cloud, also ‘bends’ the phenotypes (see Figure 4A and B). This changes the major axis angle of the fits, and thus the angle *θ*.

Additionally, in the context of RNA velocity, the upper mentioned midline is defined as the ‘steady-state line’, where the birth rate is equal to the death rate. Cells at the steady-state line—by definition of the model—have a velocity of zero. Therefore, after KNN smoothing, RNA velocities change. The loop-like shape, which is created by KNN smoothing, is the behaviour genes are expected to follow. However, in our analyses, it arises under the condition that two phenotypes with a large *θ* are smoothed ‘into’ each other, into a loop. Our finding that smoothing distorts the data is also in alignment with the conclusions of some recent studies (8,45).

Overall, while KNN smoothing may be beneficial for DGEA, it might artificially change the shape of the data. That change influences the metrics, *θ* and *ω*. Based on these observations, we conclude that it is not advisable to apply smoothing before using this shape-based analysis.

## Conclusion

The shape of bimodal scRNAseq-data is considered important, most notably in the definition of RNA velocity. Many previous studies have, for years, used this notion to gain insight into a plethora of biological processes. Our goal was to demonstrate that two-dimensional scRNA-seq data encompass more information than they are currently exploited for and to bring this implicit notion to the forefront. We showed that there are four different modalities observable and detectable in such RNA-seq data, of which DGEA detects only two. We have utilized ellipses to detect novel modalities in multiple experiments. The fits are stable, and the derived statistics, the angle *θ* and the overlap *ω*, enable detection of previously overlooked genes. Further analysis of significant genes, either DEGs or DAGs, revealed that DAGs are not always annotated in public databases with the biological processes they characterize. Because the annotation had been created using DGEA, we believe that the absence of it could imply both the novelty of these genes and the distinctness of their biological impact from that of DEGs.

While we used ellipses, other models could be considered as well. One such model is a simple linear fit. A linear model would allow for capturing the angle differences between phenotypes, similarly to the major axis of an ellipse fit; however, it would not allow for calculating the overlap statistic. Another model that could be considered is a mixed Gaussian model. A Gaussian fit would extend into a third dimension, a dimension of probabilities, over the points in a two-dimensional space. To obtain a two-dimensional representation from the Gaussian fit, this bell-shaped surface would have to be cut at a chosen probability level set, producing an ellipse. Mathematically, this would be equivalent to solving an ellipse problem but with further constraints of matching centre and covariance angle of the Gaussian. Naturally, if the data are strongly Gaussian, then the general ellipse fit would coincide with the Gaussian ellipse.

While other models may also produce useful results, we chose ellipses because of the presence of a numerical solution and their ability to encapsulate the data.

We would like to also comment on the preprocessing and the source of scRNA-seq data. First, based on our findings, KNN smoothing obscures existent shapes. Therefore, we advise against using it when applying our shape-based approach.

Fitting ellipses is not without its challenges. At lower expression levels, though possible to fit an ellipse, results are difficult to interpret. We advise against using low-expression genes, as the data are noisy, and low levels of expression do not allow for modalities to fully take shape. If such genes are of interest, this issue can sometimes be resolved by deeper sequencing. However, when generating splicing-resolved data, in some cases spliced and unspliced abundances can be difficult to quantify reliably. For example, extremely fast splicing can preclude the capture of the unspliced variant. Additionally, in 3′ biased protocols such as 10X, genes with introns far from the 3′ end are challenging to capture due to the limited reach of the sequencing. Another, more technical challenge of fitting ellipses is the influence of outliers, which is a known numerical drawback of least-squares fits. There were a few misfits observed throughout our analysis. We overcome this to a degree by cleaning the data from outliers, normalizing and bootstrapping our fits. Though effective, the latter step makes the analysis more computationally intensive. Future work could include improving the computational intensity and the robustness of the fits by further addressing the shortcomings of the least-squares approach.

Our findings indicate that there is additional information in scRNA-seq data that we refer to as ‘shape’ or ‘modality’. We hypothesize that these modalities are a result of differential regulation, perhaps in splicing. For researchers probing for biomarkers, differences in modalities can extend the search space. Furthermore, as the rate of splicing is thought to play a role in fundamental phenotypic changes, such as ageing (46), those studying such phenomena and the related regulatory processes could incorporate the shape information into their analyses.

## Supplementary Material

lqae179_Supplemental_File

## Data Availability

We used scSLAM-seq data of CRC organoids (47) (time-resolved), as available in their Supplementary data (count_matrices_slamseq.tgz, condition W), and MERFISH data capturing the cell cycle (48) (spatially resolved), as available in their Supplementary datasets S12 and S14. Source code is available at https://git.zib.de/sunkara/ellipses-for-transcription-modality-discovery.
