# Supplementary material for: Exploring transcription modalities from bimodal, single-cell RNA sequencing data
Source: NAR Genom Bioinform. 2024 Dec 18;6(4):lqae179. doi: 10.1093/nargab/lqae179 (PMC11655292; doi:10.1093/nargab/lqae179)
Supplement: lqae179_Supplemental_File [file lqae179_supplemental_file.pdf]

Exploring transcription modalities from bimodal, single-cell RNA sequencing data

December 10, 2024

1 Supplementary Figures S1-S7

References

[1] Arthur Liberzon, Aravind Subramanian, Reid Pinchback, Helga Thorvaldsdóttir, Pablo Tamayo, and Jill P Mesirov. Molecular signatures database (msigdb) 3.0. *Bioinformatics*, 27(12):1739–1740, 2011.

List of Figures

1 **No batch effect is observable in the CC dataset**. a-b) Library normalised, scaled and log transformed expression heatmaps of batch 1 marked in green in the column header, batch 2 in yellow, and batch 3 in pink. Shared legend below the figures. a) Columns clustered. b) Columns sorted by batch. Batches do not cluster together. c) Venn diagram of the 50 most significant genes resulting from independently performed DGEA on each batch, and on all cells (Merged). The results largely overlap. 5

2 **Results of GO enrichment in recurrent DEGs of the CC dataset.** Y axis: significant GO terms, X axis: false discovery rate (FDR) values corresponding to significant GO terms. GO terms are nested and the level of hierarchy is indicated by the length of those arrows before a term. GO terms of the same branch are highlighted by the light colour bands in the background. 6

3 **GSEA results of genes ranked by DGEA in patient 9 in the CRC dataset.** Y axis labels are gene set names as listed on the GSEA website or the MSigDB [1], bars over the X axis measure the FDR of significant gene sets. Positive enrichment, meaning that the gene set tends to be upregulated in CRC patient 9, is indicated yellow coloured bars, while negative enrichment with blue coloured bars. 7

4 **GSEA results of genes ranked by DGEA in patient 13 in the CRC dataset.** Y axis labels are gene set names as listed on the GSEA website or the MSigDB [1], bars over the X axis measure the FDR of significant gene sets. Positive enrichment, meaning that the gene set tends to be upregulated in CRC patient 13, is indicated by yellow coloured bars, while negative enrichment by blue coloured bars. 8

5 **Phase plots of example DAGs.** a) Phase plots of genes *ZNF404*, *POTEI*, and *DDX39B* and the corresponding ellipse fits in solid lines of the pooled data (all batches) in the cell cycle dataset. Green: cells in phase S, red: cells in phase G2M. b) Phase plots of genes *MT-ND1*, *MT-ND3*, and *TXN* and the corresponding ellipse fits in solid lines of patient 13 in the colorectal cancer dataset. Blue: healthy cells, red: cancerous cells. 9

6 **Root Mean Square Deviation (RMSD) of ellipse fits versus the mean expression of genes.** The RMSD is scaled by the major axis length. Mean expression = 10 is marked by a solid red line. a) RMSD plots of the cell cycle dataset batches 1-3 as well as of the pooled data ("All batches"). b) RMSD plots of the colorectal cancer dataset, patients 009 and 013. 10

7 **Comparison of ellipse fitting approaches: freely fitted vs. forcing the major axis through the origin** This figure demonstrates the two implemented ellipse-fitting approaches in the colorectal cancer dataset (patient 009) using the gene NME1-NME2 as an example. The x and y axes represent old and new transcript levels, respectively. The first panel shows ellipses fit freely to minimize the RMSD, while the second panel displays ellipses fit with the major axis forced to pass through the origin. The corresponding angles, RMSD values, and differences between conditions are displayed above the corresponding figures for comparison. 11

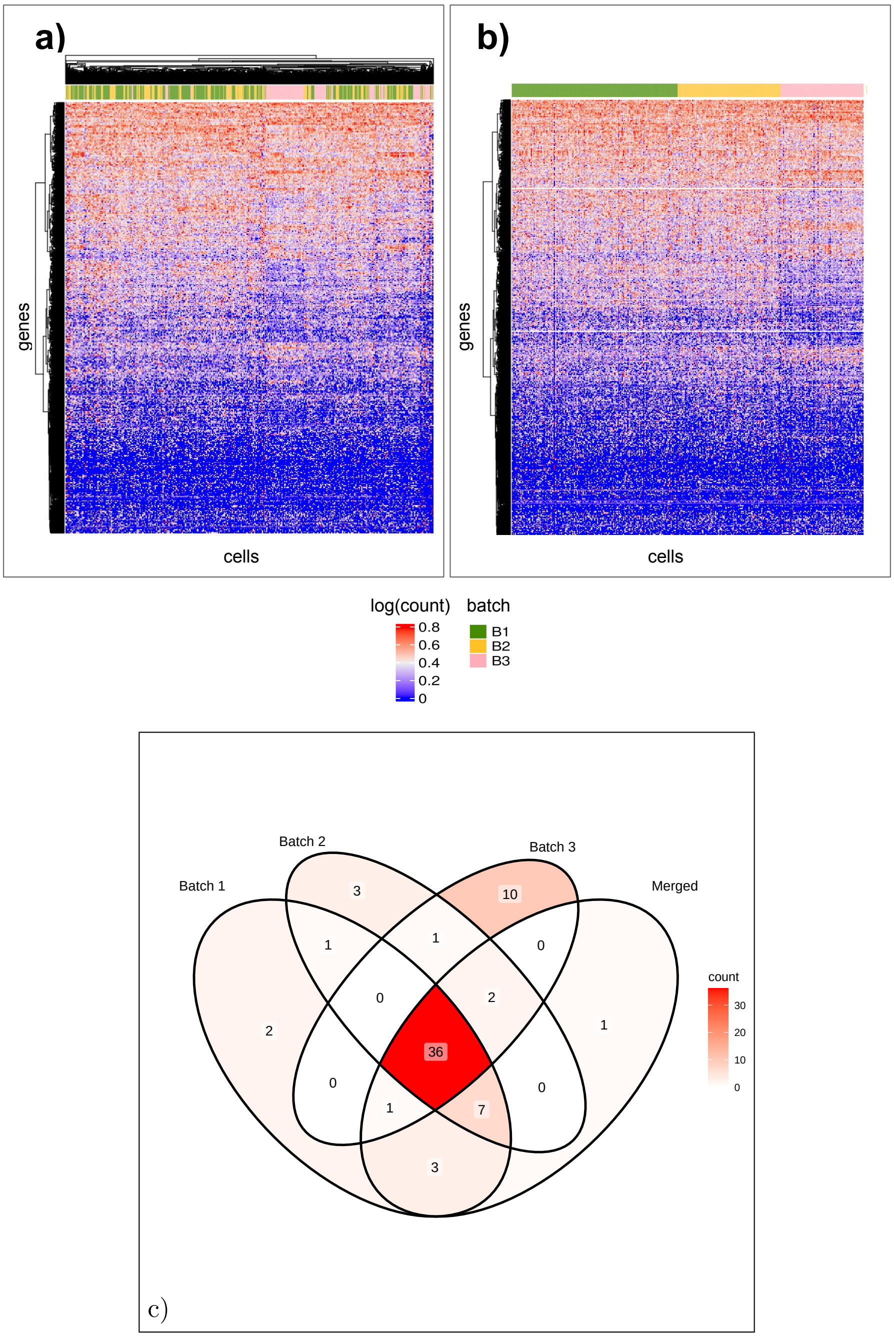

Supplementary Figure 1 – **No batch effect is observable in the CC dataset**. a-b) Library normalised, scaled and log transformed expression heatmaps of batch 1 marked in green in the column header, batch 2 in yellow, and batch 3 in pink. Shared legend below the figures. a) Columns clustered. b) Columns sorted by batch. Batches do not cluster together. c) Venn diagram of the 50 most significant genes resulting from independently performed DGEA on each batch, and on all cells (Merged). The results largely overlap.

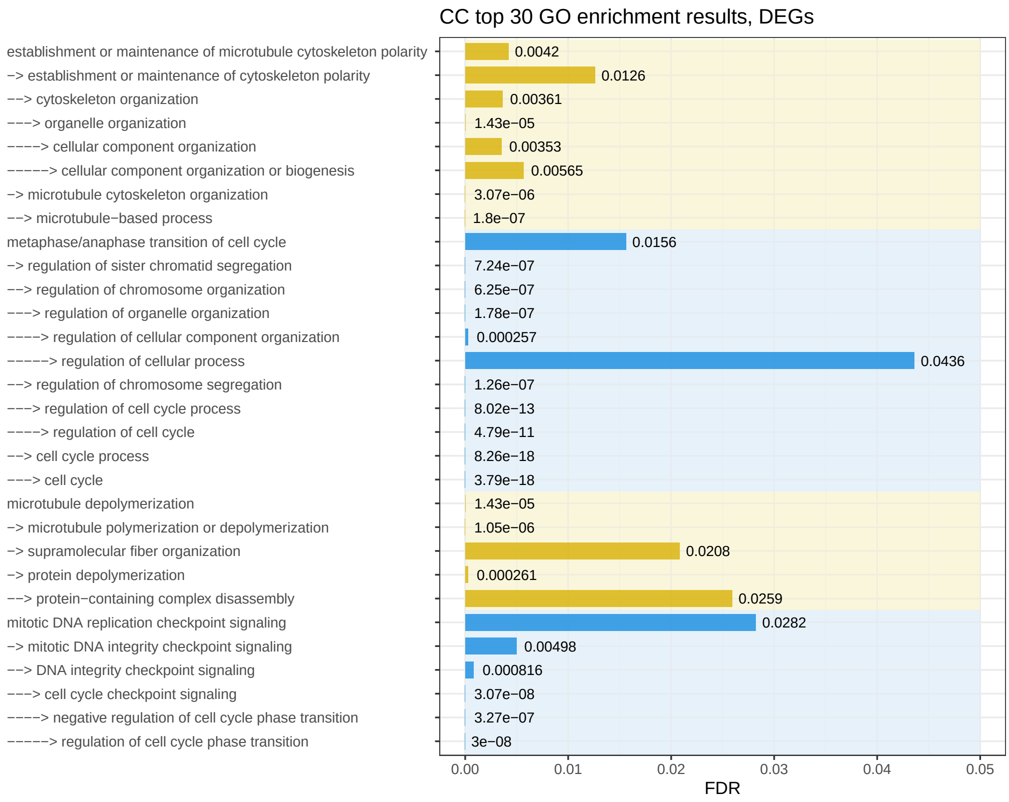

Supplementary Figure 2 – **Results of GO enrichment in recurrent DEGs of the CC dataset.** Y axis: significant GO terms, X axis: false discovery rate (FDR) values corresponding to significant GO terms. GO terms are nested and the level of hierarchy is indicated by the length of those arrows before a term. GO terms of the same branch are highlighted by the light colour bands in the background.

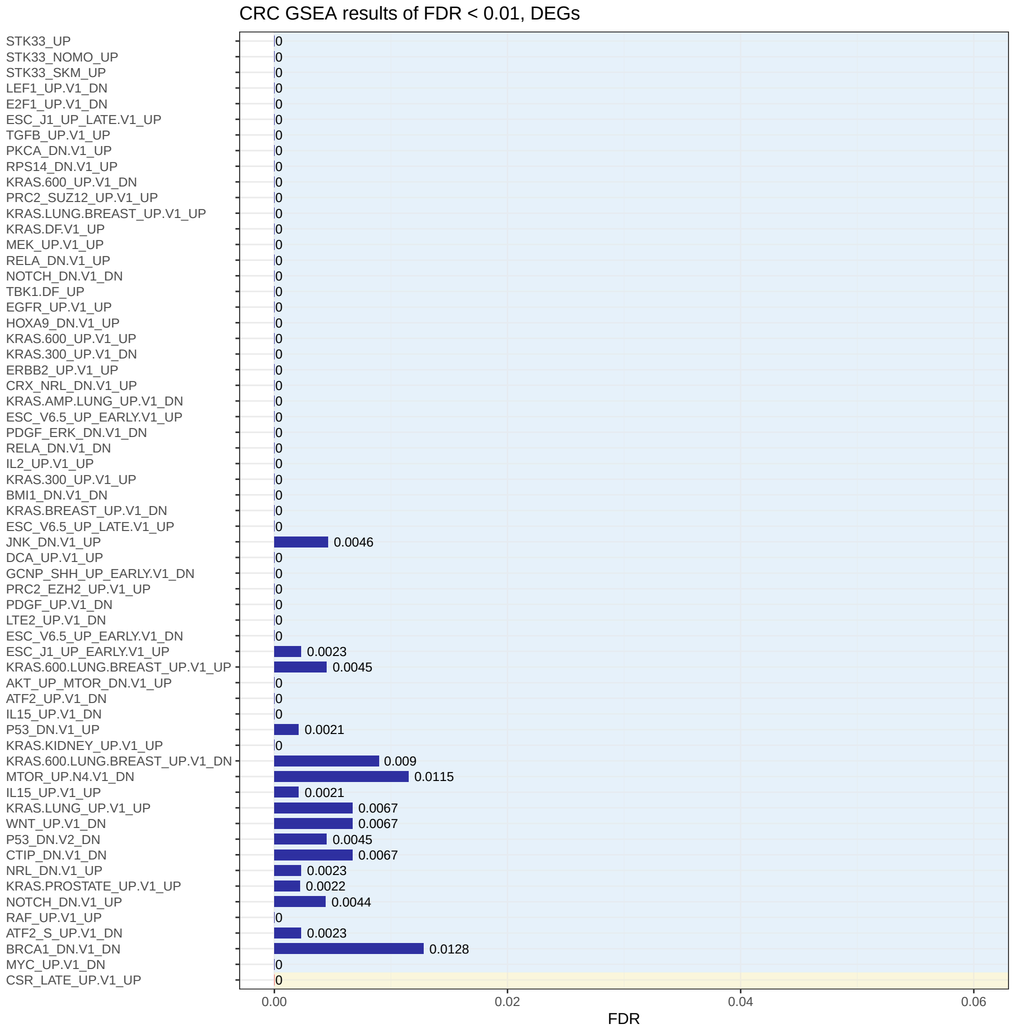

Supplementary Figure 3 – **GSEA results of genes ranked by DGEA in patient 9 in the CRC dataset.** Y axis labels are gene set names as listed on the GSEA website or the MSigDB [1], bars over the X axis measure the FDR of significant gene sets. Positive enrichment, meaning that the gene set tends to be upregulated in CRC patient 9, is indicated yellow coloured bars, while negative enrichment with blue coloured bars.

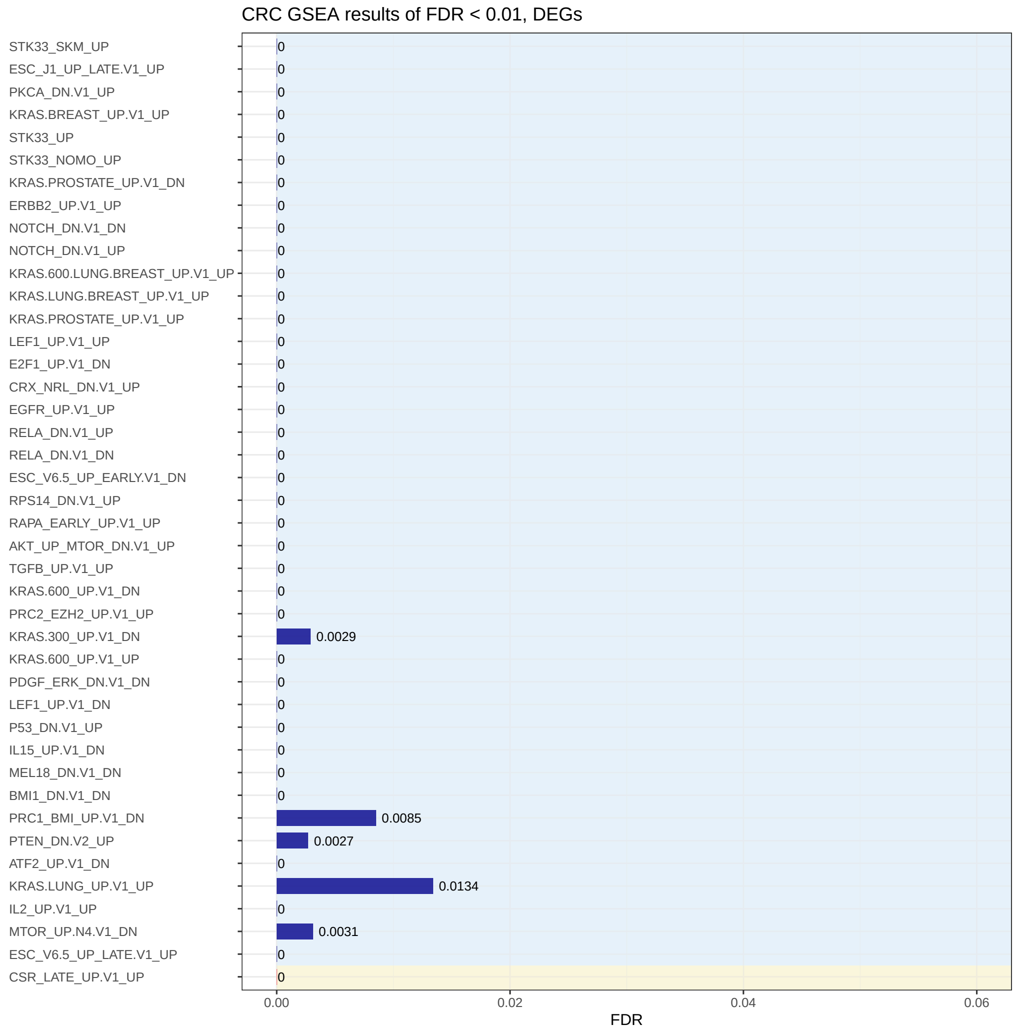

Supplementary Figure 4 – **GSEA results of genes ranked by DGEA in patient 13 in the CRC dataset.** Y axis labels are gene set names as listed on the GSEA website or the MSigDB [1], bars over the X axis measure the FDR of significant gene sets. Positive enrichment, meaning that the gene set tends to be upregulated in CRC patient 13, is indicated by yellow coloured bars, while negative enrichment by blue coloured bars.

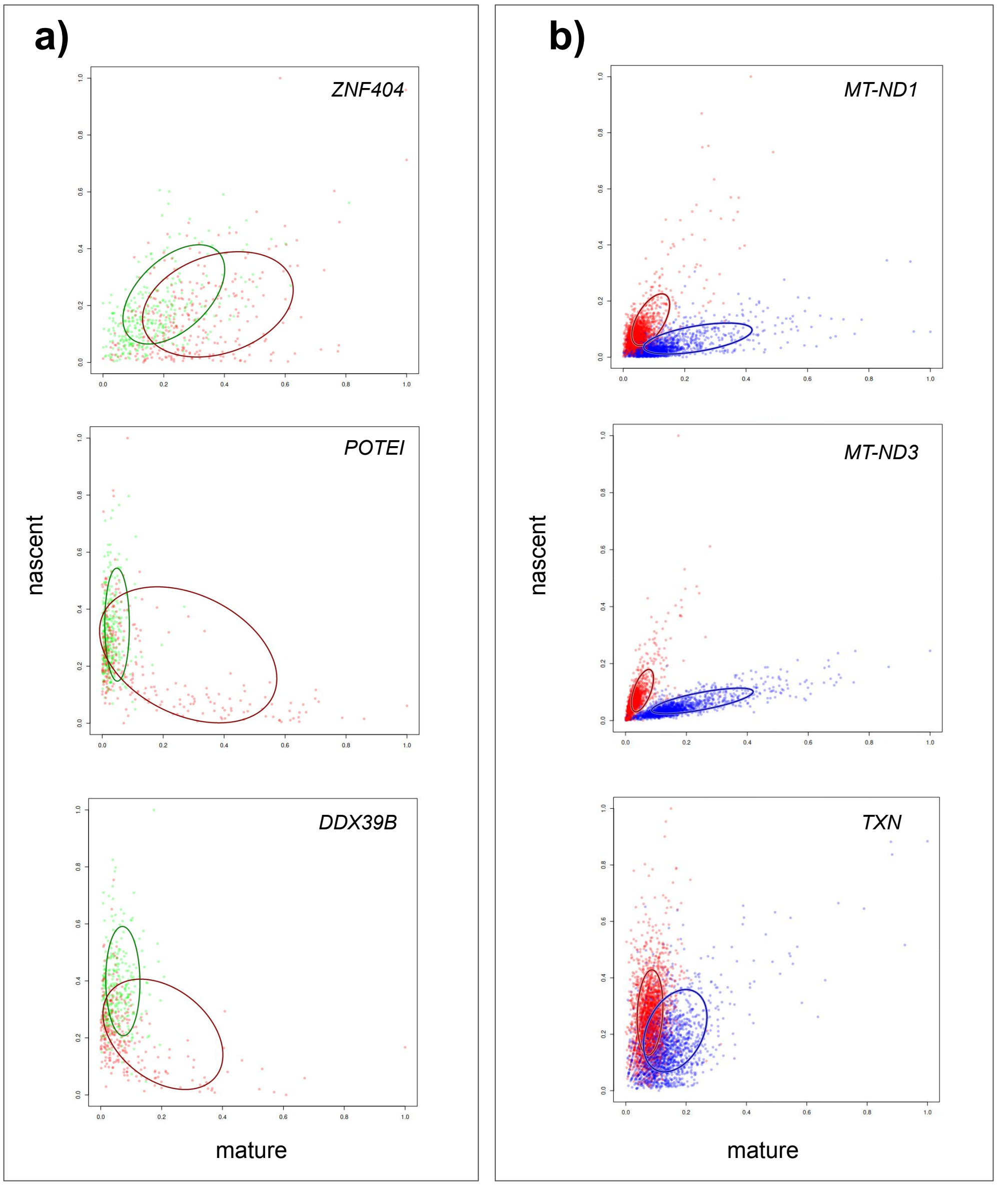

Supplementary Figure 5 – **Phase plots of example DAGs.** a) Phase plots of genes *ZNF404*, *POTEI*, and *DDX39B* and the corresponding ellipse fits in solid lines of the pooled data (all batches) in the cell cycle dataset. Green: cells in phase S, red: cells in phase G2M. b) Phase plots of genes *MT-ND1*, *MT-ND3*, and *TXN* and the corresponding ellipse fits in solid lines of patient 13 in the colorectal cancer dataset. Blue: healthy cells, red: cancerous cells.

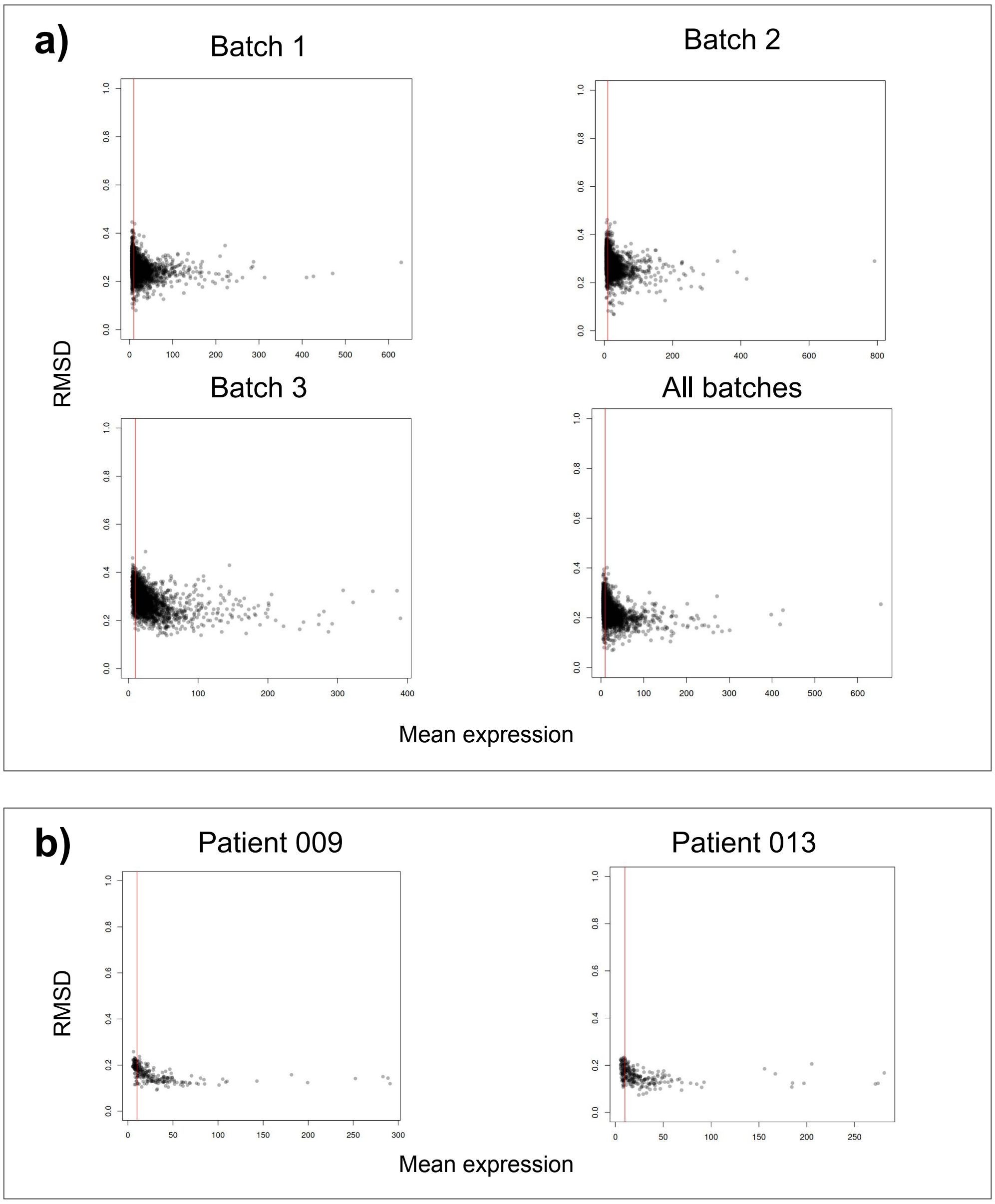

Supplementary Figure 6 – **Root Mean Square Deviation (RMSD) of ellipse fits versus the mean expression of genes.** The RMSD is scaled by the major axis length. Mean expression = 10 is marked by a solid red line. a) RMSD plots of the cell cycle dataset batches 1-3 as well as of the pooled data ("All batches"). b) RMSD plots of the colorectal cancer dataset, patients 009 and 013.

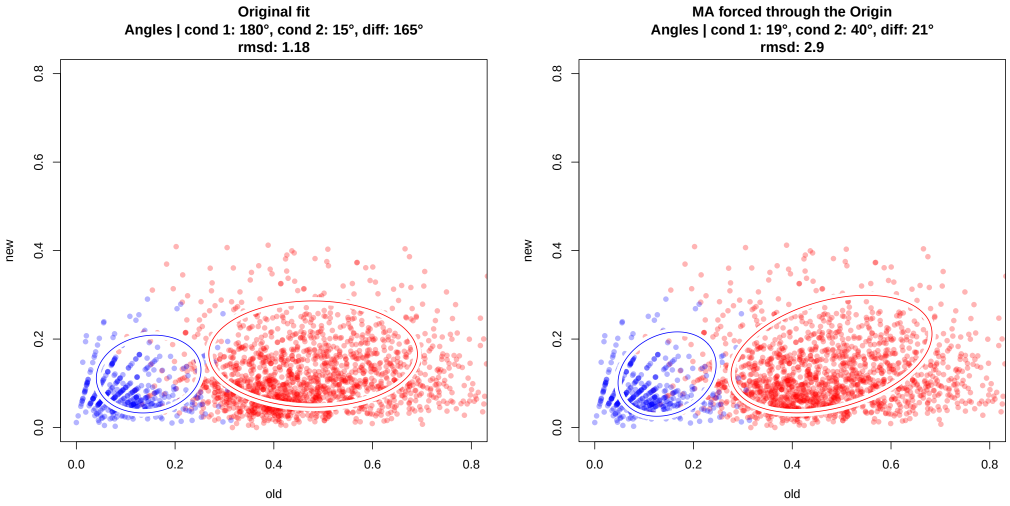

Supplementary Figure 7 – **Comparison of ellipse fitting approaches: freely fitted vs. forcing the major axis through the origin** This figure demonstrates the two implemented ellipse-fitting approaches in the colorectal cancer dataset (patient 009) using the gene NME1-NME2 as an example. The x and y axes represent old and new transcript levels, respectively. The first panel shows ellipses fit freely to minimize the RMSD, while the second panel displays ellipses fit with the major axis forced to pass through the origin. The corresponding angles, RMSD values, and differences between conditions are displayed above the corresponding figures for comparison.
